# Dataset on the power dissipated by rolling resistance of a prototype photovoltaic electric vehicle while varying the mass

**DOI:** 10.1016/j.dib.2023.109957

**Published:** 2024-01-03

**Authors:** Sidy Mactar Sokhna, Mohamed El Amine Ait Ali, Souleye Faye, Vincent Sambou, Mohamed Agouzoul

**Affiliations:** a Laboratoire Eau, Energie, Environnement et Procédés Industriels (LE3PI), École Supérieure Polytechnique de Dakar, BP: 5085, Dakar-Fann, Sénégal; b Université Mohammed V de Rabat, Ecole Mohammadia d'Ingénieurs, Équipe de Recherche Génie Mécanique et Énergétique : Modélisation et Expérimentation ERG2(ME) Av. Ibn Sina B.P. 765, Rabat, Morocco

**Keywords:** Dynamic equation, Friction, Energy, Coefficient of traction

## Abstract

The examined vehicle is an electric generator fuelled by a photovoltaic source. A portion of this energy is allocated for mobility at a top speed of 35.7 km/h, covering a distance of 70 km and the mass of this electrical vehicle is 300 kg. Another portion is collected and transformed into electricity for domestic use. For propulsion, electric motors with 3000 W nominal power and a voltage of 48 V are situated in the rear wheels. Part of this power is lost due to the resistance like the air resistance, the rolling resistance and the resistance due to the slope. All the resistances must be overcome during travel, with the majority of this resistance attributed to rolling resistance. These data can be used to quantify the power dissipated by the rolling resistance of the generator, which is an electric vehicle. A first experiment was conducted using an electric vehicle protocol to gather data, which allowed for recording of various speeds and corresponding starting powers. The gathered data was then used to determine the rolling resistance value by employing a resolution method that considers factors such as tyre inflation pressure, speed, and vehicle mass. This method involves calculating a coefficient known as the traction coefficient, which arises from the tyre's local deformation under wheel load. The power dissipated by rolling resistance can be determined from this data. Using MATLAB, we can visualise the variations in power dissipated by the rolling resistance from the data. The vehicle is travelling on a surface made of asphalt concrete. This study shows the behaviour of electric vehicles and will help to determine their performance under driving conditions, taking into account rolling resistance, mass and speed.

Specifications Table

**Table:** 

Subject	Energy, Engineering
Specific subject area	Energy Engineering and Power Technology
Data format	Raw, Analyzed
Type of data	Table, Image, Chart, Graph, Figure
Data collection	Thegathereddatadisplaystheinitialpowersatvariousspeeds.Thedatawasobtained fromthedashboards,connectetothedrivewheels,andcontrollers,whichprovide informationontheobservedparameters.Priortotesting,thevoltageofeachbatterycell hasbeenmeasuredat3.2V.Astheyareconnectedinseries,thetotalvoltageoutputiscalculatedbysummingtheindividualvoltagevalues,systemvoltagewasmeasuredusinga multimeterandrecordedas51.3V.Solarradiationwasmeasured.Thedatawasthen presentedingraphformusingExcelandMATLAB.
Data source location	The observations were made at the Ecole Supérieure Polytechnique de Dakar in Senegal, with geographical coordinates of longitude 17.43°, latitude 14.71°, and an average daily solar radiation of 481 W/m^2^.
Data accessibility	Repository name: Mendeley Data Data identification number: DOI: 10.17632/dsm8j3fhck.1 Direct URL to data: https://data.mendeley.com/datasets/dsm8j3fhck/1

## 1 Value of the Data


 

• As the vehicle is fully powered by photovoltaic cells and marks the first of its kind to be built in Senegal, it is essential to characterise this vehicle using these specific data which are the mass, the tyre inflation pressure and the speed. The data obtain in the first test is important to observe the electric vehicle's performance under test conditions in the Sahel region.
• These data will be valuable for the scientific community, particularly those seeking to understand the behaviour of electric vehicles, specifically those powered by photovoltaic energy.
• These data can be used by other researchers who wish to study the conditions of electric vehicle movement, taking into account rolling resistance, mass, speed, and power variation.
• This data will aid in characterising an electric vehicle and energy modelling by factoring in the power lost through rolling resistance that affects the battery's charge level, alongside vehicle travel conditions.
• They also pointed out to the Scientific Committee the importance of taking into account the inflation pressure of wheels with integrated brushless direct current (BLDC) motors and the speed when calculating the rolling resistance using a coefficient K, commonly referred to as the traction coefficient in the Andreau formula, which provides a more accurate estimate of the rolling resistance.

## 2 Background

Electric cars can be more efficient if they use photovoltaic energy as a source, depending on the driving situation. Since a large proportion of energy is produced from fossil fuels, the use of electrical vehicles as generators of electrical energy enables a switch to renewable sources. A variety of techniques are employed to estimate rolling resistance [1], [2], [3], [4]. The numerical method is frequently used [5]. Employing static finite elements [6] is an alternative. One method [7,8] is to use an ADIS 16385 measuring device. This software is utilised for ascertaining the kinematic traits of vehicle motion. In this method, the time describing the descent is the time during which the speed drops from 10% of its maximum speed. Tyres are subjected to tests and the rolling resistance value is determined using another technique [[9], [10], [11]]. Alongside the test conditions, data is necessary to determine the amount of power lost through rolling resistance while driving. The rolling resistance method considers vital parameters, like speed and wheel inflation pressure. The data employed in this study banks on the efficiency of BLDC engines.

## 3 Data Description

The dataset contains the battery voltage, which was measured at 51.3 V, and is comprised of 16 cells, each producing 3.2 V. Additionally, the average solar irradiation per hour in the day measures 481 W/m^2^, the average duration of irradiation is 9 hours and measurements were taken at an ambient temperature of 27 °C. The BLDC wheel motors have both a peak and rated power of 6000 W and 3000 V, respectively. The motor current stands at 8 A, while the observed speed is 560 rpm with 85% efficiency. There is also a range of nine different linear speeds provided in km/h, which are as follows: 15, 18, 21, 24, 27, 30, 33, 35, and 35.7. For each velocity, there is an associated initial power of 381 W, 361 W, 299 W, 230 W, 362 W, 758 W, 438 W, 604 W, and 783 W, respectively. The rolling resistance data reveals that 31.90 N; 42.54 N; 53.17 N; and 106.35 N are obtained for each mass applied at maximum speed. According to the data, the corresponding power losses are 372.23 W; 496.31 W; 620.38 W; and 1240 W respectively. Wheel inflation pressure is 3 bar.

Table 1 gives the rolling resistance value which has an average value of 31.9 N for an unladen mass of 300 kg. Table 2 gives an average rolling resistance value of 42.54 N for a mass of 400 kg, Table 3 gives a rolling resistance value of 53.17 N for a mass of 500 kg and Table 4 gives a rolling resistance value of 106.35 N for a mass of 1000 kg. The mass of the conductor is 75 kg and is included in the total mass (Table 5, Table 6, Table 7, Table 8).

**Table 1:** Variation of rolling resistance for *m* = 300 kg.

Speed *(km/h)*	Traction coefficient K	Rolling resistance*(N)*
15	99.30	29.70
18	105.95	31.78
21	105.97	31.79
24	106.01	31.80
27	106.07	31.82
30	106.14	31.84
33	106.24	31.87
35	106.32	31.89
35.7	106.35	31.90

**Table 2:** Variation of rolling resistance for *m =* 400 kg.

Speed *(km/h)*	Traction coefficient K	Rolling resistance*(N)*
15	99.30	39.61
18	105.95	42.38
21	105.97	42.39
24	106.01	42.40
27	106.07	42.43
30	106.14	42.46
33	106.24	42.50
35	106.32	42.53
35.7	106.35	42.54

**Table 3:** Variation of rolling resistance for *m =* 500 kg.

Speed *(km/h)*	Traction coefficient K	Rolling resistance*(N)*
15	99.30	49.51
18	105.95	52.97
21	105.97	52.98
24	106.01	53.00
27	106.07	53.03
30	106.14	53.07
33	106.24	53.12
35	106.32	53.16
35.7	106.35	53.17

**Table 4:** Variation of rolling resistance for *m =* 1000 kg.

Speed *(km/h)*	Traction coefficient K	Rolling resistance*(N)*
15	99.30	99.30
18	105.95	105.95
21	105.97	105.97
24	106.01	106.01
27	106.07	106.07
30	106.14	106.14
33	106.24	106.24
35	106.32	106.32
35.7	106.35	106.35

**Table 5:** Power variation for *m =* 300 kg.

Speed *(km/h)*	Rolling resistance*(N)*	Power (W)
15	29.70	145.63
18	31.78	186.97
21	31.79	218.18
24	31.80	249.45
27	31.82	280.77
30	31.84	312.19
33	31.87	343.72
35	31.89	364.83
35.7	31.90	372.23

**Table 6:** Power variation for *m =* 400 kg.

Speed *(km/h)*	Rolling resistance*(N)*	Power (W)
15	39.61	191.17
18	42.38	249.30
21	42.39	290.91
24	42.40	332.59
27	42.43	374.36
30	42.46	416.25
33	42.50	458.30
35	42.53	486.44
35.7	42.54	496.31

**Table 7:** Power variation for *m =* 500 kg.

Speed *(km/h)*	Rolling resistance*(N)*	Power (W)
15	49.51	242.72
18	52.97	311.61
21	52.98	363.65
24	53.00	415.75
27	53.03	467.96
30	53.07	520.31
33	53.12	572.87
35	53.16	608.04
35.7	53.17	620.38

**Table 8:** Power variation for m =1000 kg.

Speed *(km/h)*	Rolling resistance*(N)*	Power (W)
15	49.51	485.44
18	52.97	623.23
21	52.98	727.29
24	53.00	831.45
27	53.03	935.91
30	53.07	1040.63
33	53.12	1145.74
35	53.16	1216.09
35.7	53.17	1240.77

The above graphs illustrate the changes in rolling resistance values according to applied mass and speed. The results show that rolling resistance is dependent on mass and minimally affected by speed. Additionally, a 100 kg increase in mass, beyond the weight of the electric vehicle, causes an average increase in rolling resistance of 12 N.

Rolling resistance is not only a tire property but also a pavement property and must be addressed to reduce energy [12]. In our case, the vehicle is travelling on a concrete asphalt surface. Concrete asphalt consist of mineral aggregate bound together with bitumen, laid in layers and compated [13]. The tables present the values for Power dissipated by rolling resistance for different masses. At the maximum stabilized speed, we notice that Power dissipated increases by 124 W. For a mass of 300 kg, the Power dissipated is 372.23 W, for 400 kg it is observed to be 496.31 W, and for a mass of 500 kg it is 620.28 W. For a mass of 1000 kg, the power dissipated by rolling resistance is 1240.77 W. These observations are plotted as variation curves in the following graphs, one for each applied mass.

## 4 Experimental Design, Materials and Methods

### 4.1 Experimental design and materials

**Fig. 1:**
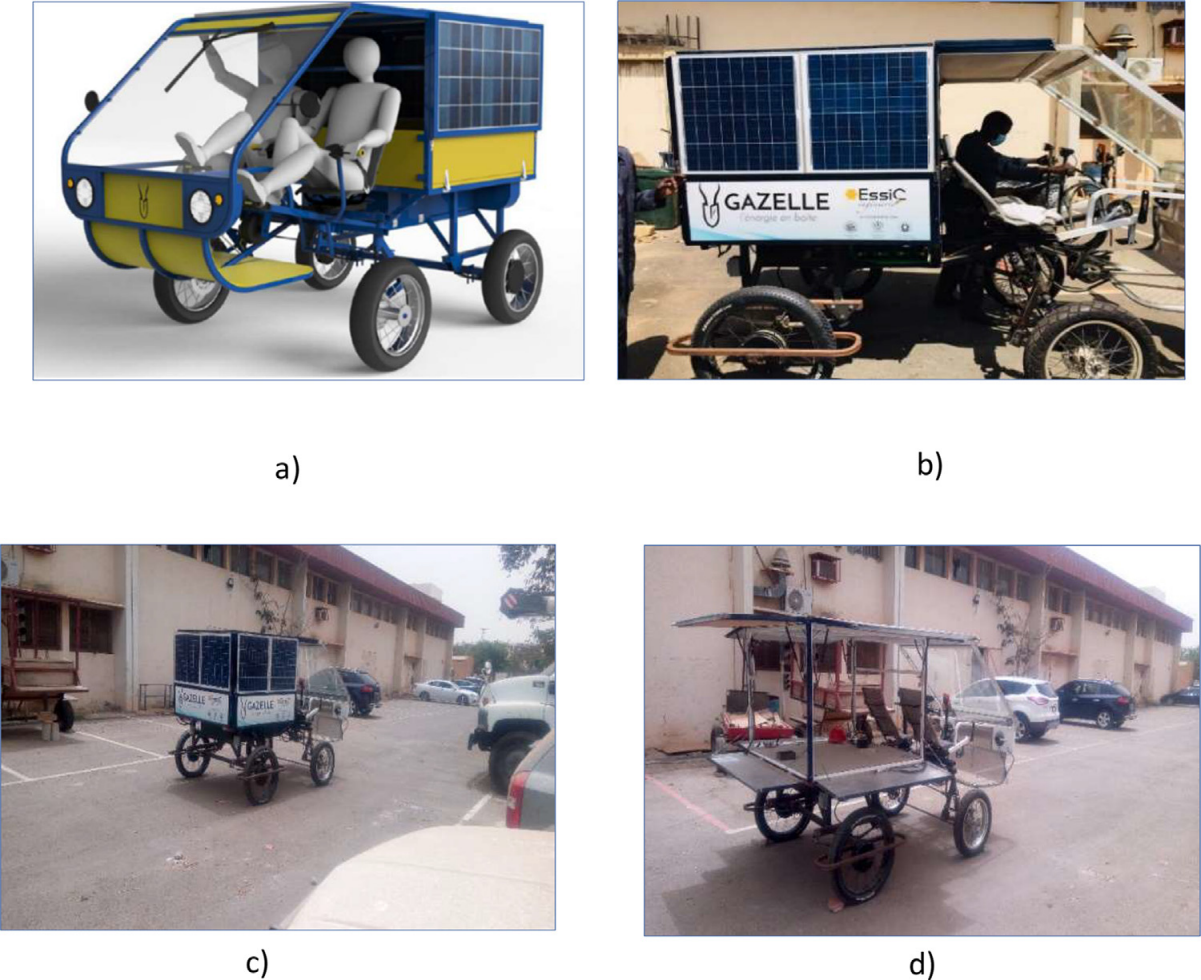
a) 3D view of the vehicle; b) vehicle produced; c) moving vehicle photo; d) Deployed vehicle panels.

**Fig. 2:**
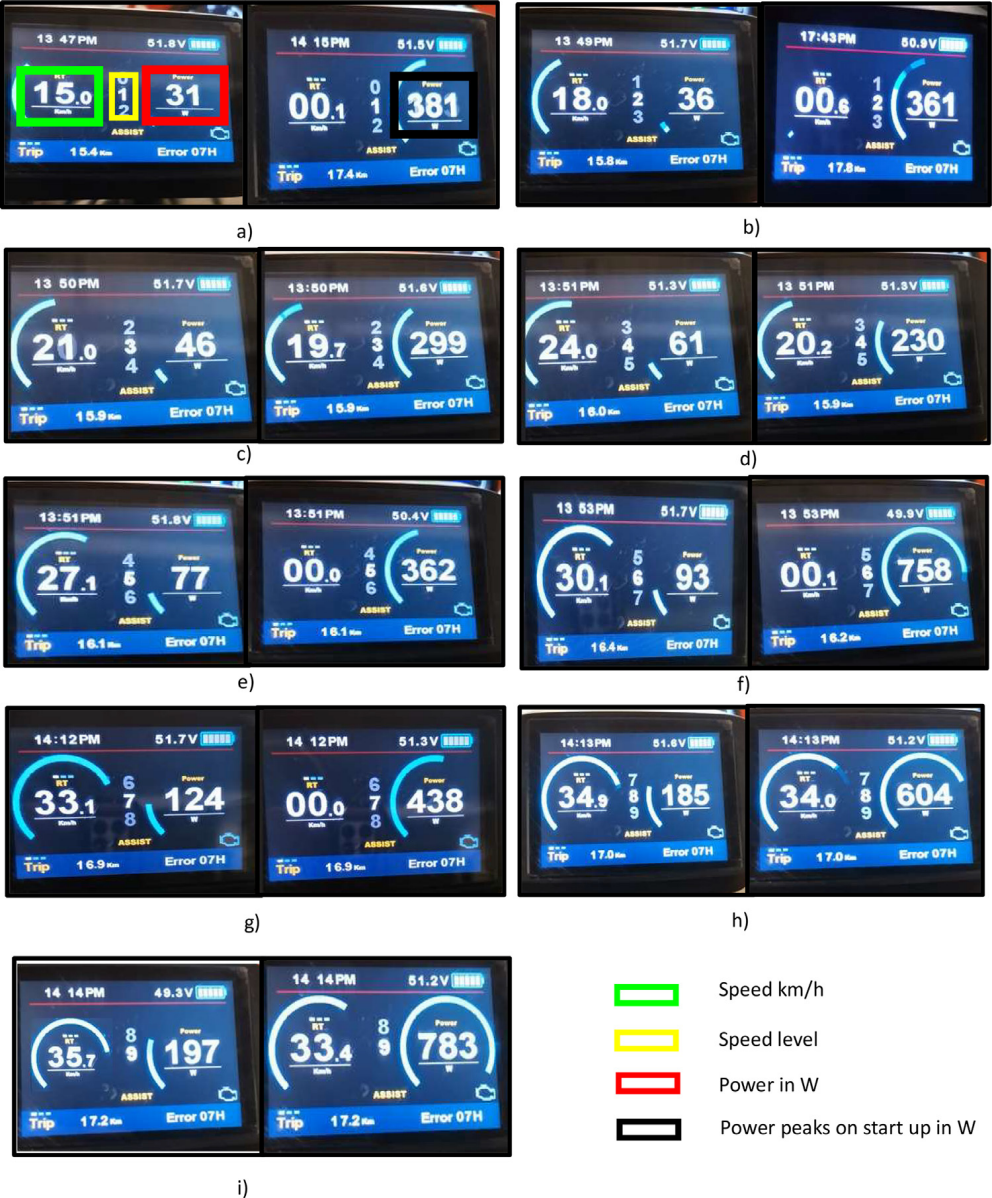
From a to j variation in power peaks on start-up and rated power as a function of speed.

**Fig. 3:**
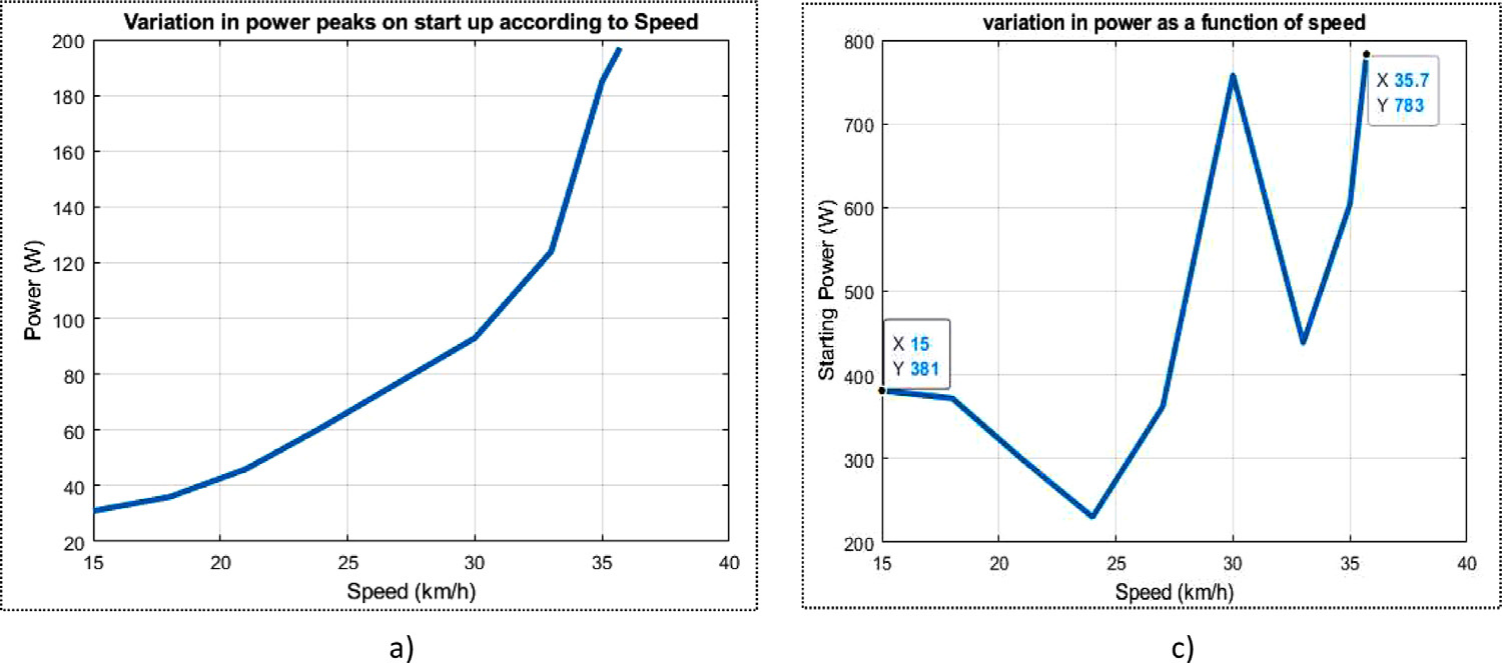
a) Variation of rated power b) power peaks on start up.

**Fig. 4:**
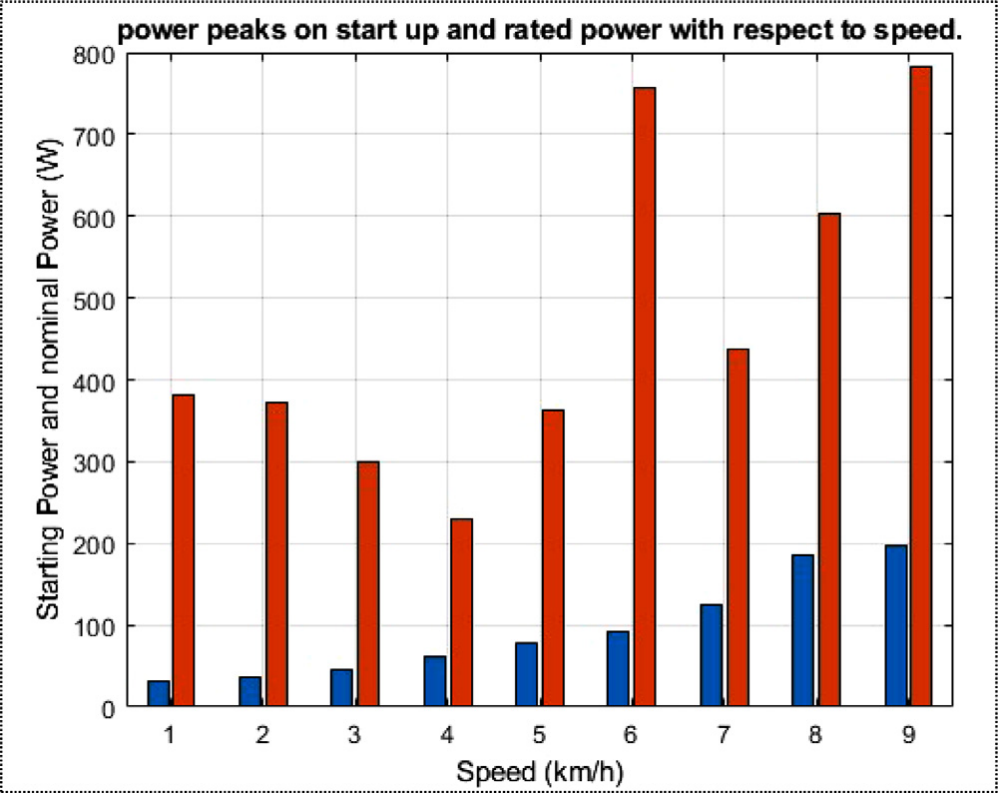
Power peaks on start-up and rated power.

**Fig. 5:**
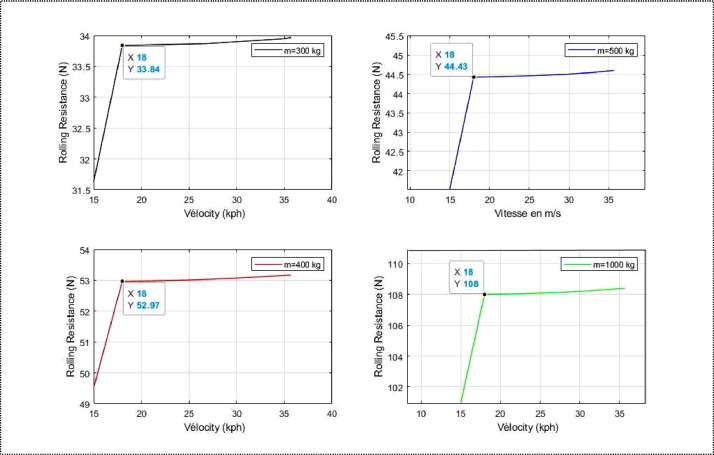
Variation of rolling resistance as function of speed.

**Fig. 6:**
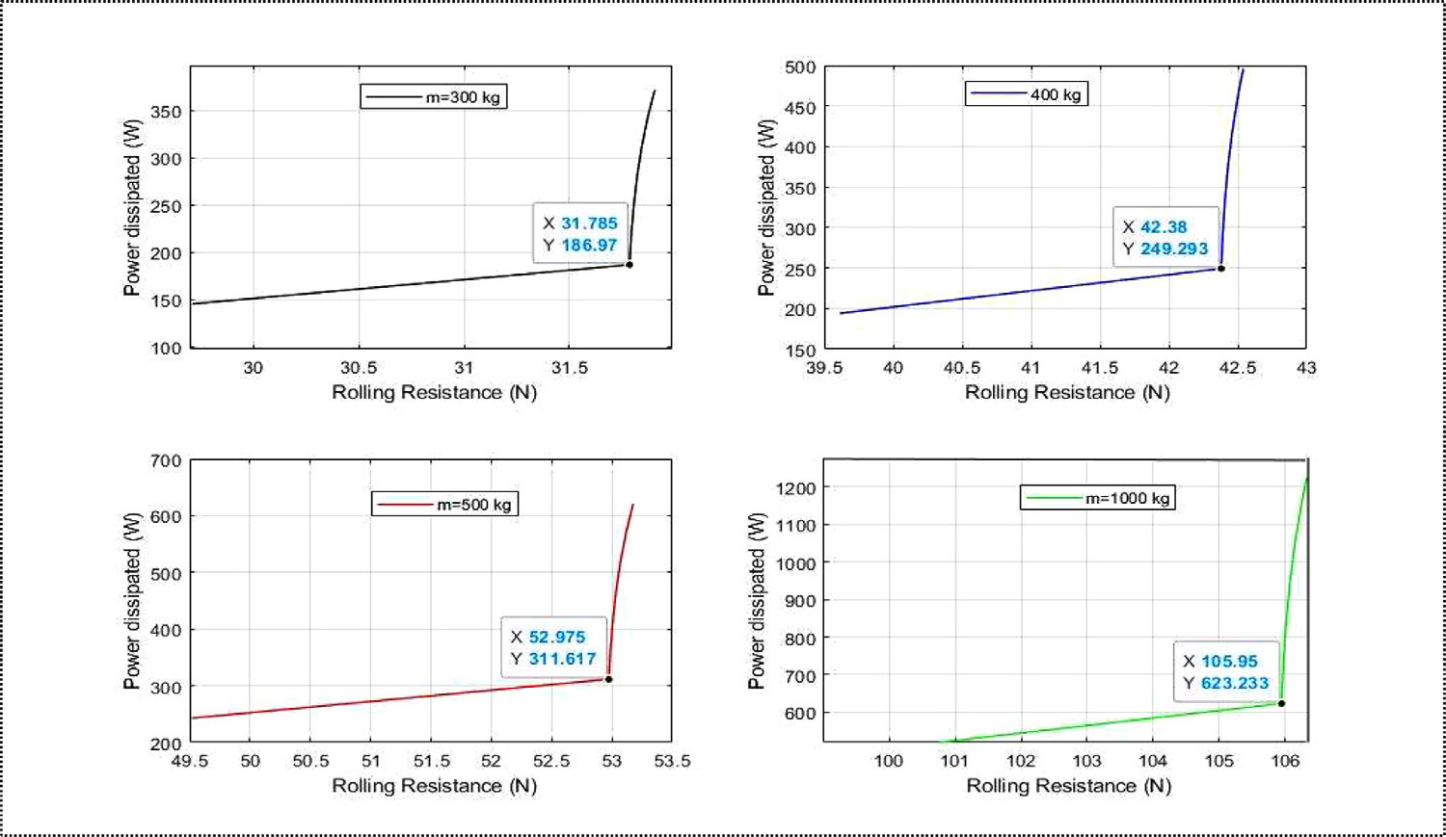
Power dissipated as function of rolling resistance.

**Fig. 7:**
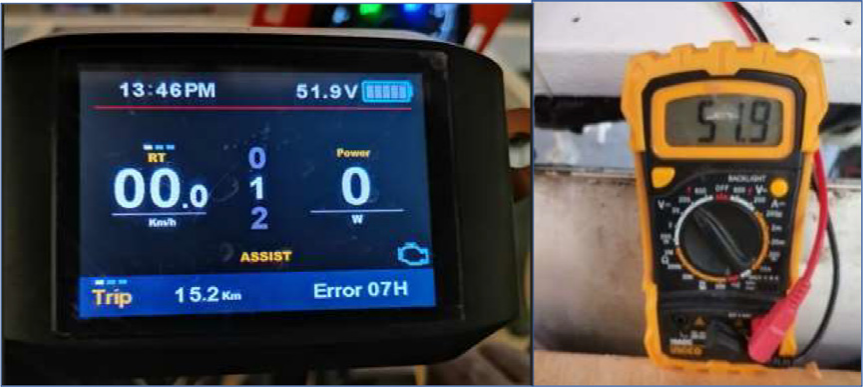
System voltage.

Different techniques can be utilised to compute rolling resistance as indicated in background. To acquire data, we utilized a multimeter to measure the initial system voltage, which was 51.9 V, as seen in the figure. Moreover, we gauged the terminal voltage of each cell, resulting in 3.2 V. We connected two electric bike meters to the motors to obtain crucial information about the 9-step speed selection, starting power, and rated power. These meters also provided us with the system voltage (Fig. 1, Fig. 2, Fig. 3, Fig. 4, Fig. 5, Fig. 6, Fig. 7).

**Fig. 8:**
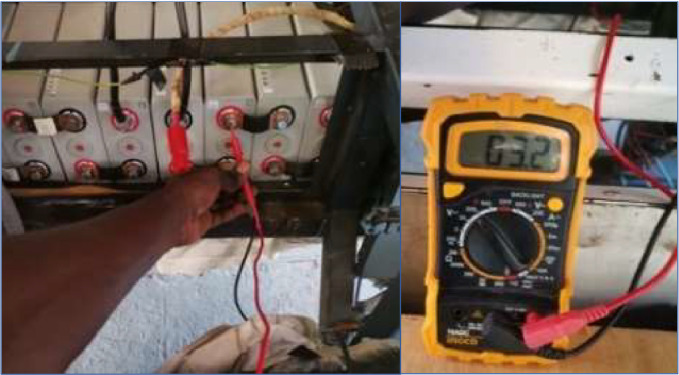
Cell voltage.

**Fig. 10:**
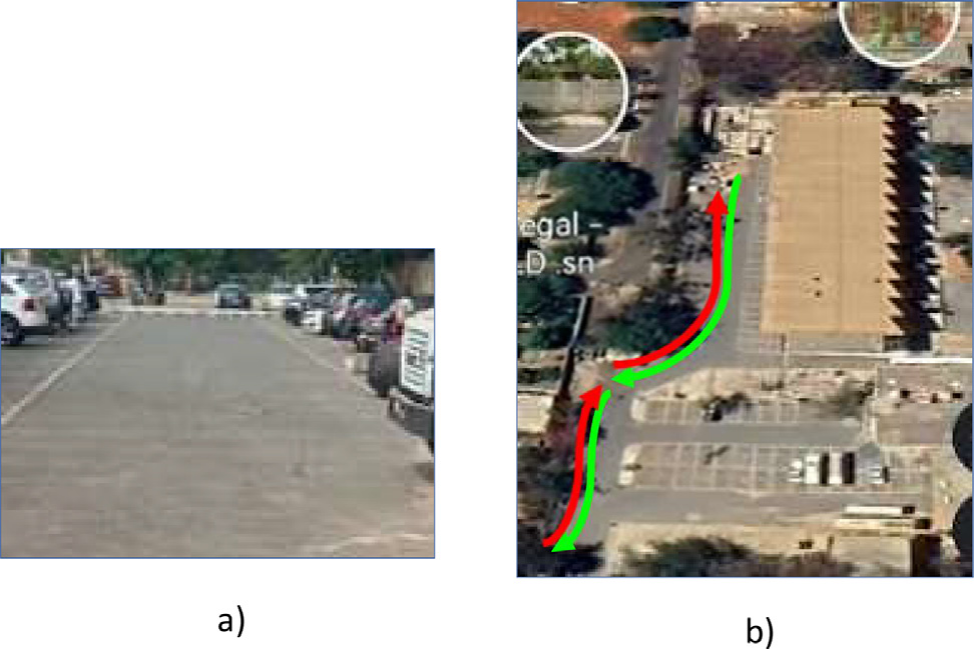
(a) Flat surface of the test route; b) Itinerary round trip .

To ensure a thorough and precise test, a full experimental protocol was followed. Initially, the power terminals of the electric motors were connected to the battery, which was linked in a series to obtain a 48 V voltage that propels the motors found in the rear wheels of the vehicle. Next, a comprehensive examination of all mechanical systems was conducted, which included the suspension, brakes and steering. Additionally, the ambient temperature and solar irradiation were recorded. The procedure was executed to guarantee a full and impartial experience. System and battery cell voltages were measured with a multimeter, which can be seen in Fig. 8. The vehicle was initiated with the ignition key before adjusting the accelerator by hand for the acceleration data. Utilize the button on the display screen to change the speed. The experiments were conducted on a flat surface composed of asphalt concrete, a blend of sand, gravel, and a petroleum by product as illustrate on Fig. 10 – a). This surface contains minimal gaps. The route covers a distance of 800 m for the outward journey and 800 m for the return journey, making the total distance 1.6 km as illustrate on Fig. 10 - b). Itinerary is the car park of the “Ecole Supérieure Polytechnique de Dakar” at Cheikh Anta Diop University. The test commences at 1:46 pm and concludes at 2:14 pm, resulting in a duration of 28 min.

**Fig. 9:**
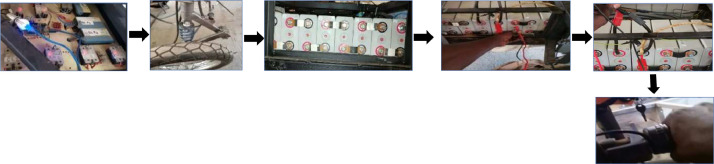
Test phase for electric vehicle start-up.

To summarise the steps in Fig. 9, we have:

- Check the voltage of the battery
- Connect the motors to the battery
- Inspect the mechanical systems including the suspension, brakes, and steering
- Initiate the start-up process
- Change gears
- Test acceleration
- Record power readings.

### 4.2 Methods

To establish the rolling resistance, we shall introduce the traction coefficient K, which considers the fluctuating speed and the tyre inflation pressure fixed at 3 bar. The Traction Coefficient K, calculated using Andreau's formula (Eq. (1)), establishes a coefficient as a function of speed and inflation pressure. This Eq. (1) is dependent on the mass, thus we alter the vehicle's mass accordingly 300 kg, 400 kg, 500 kg and 1000 kg mass. The driver weighs 75 kg. For each mass considered as a variable parameter, K is calculated as a constant taking into account the inflation pressure and at the given speed varying from 15 to 35.7 km/h with a constant parameter which is the inflation pressure.

Power is calculated in watts according to the Eq. (3), and its variation for different generator loads is determined using a function of rolling resistance.

The impact of rolling resistance and mass on power dissipation is analysed to determine which parameter primarily influences motors power at a maximum speed of 35.7 km/h.

After the necessary data has been, the rolling resistance is determined. The formula involves calculating the traction coefficient K using Eq. (2) that account for tyre inflation pressure and speed. The value of the rolling resistance allows for the determination of power. The mass is given in tonnes using this formula.

(1) $$R_{r}=M\times K$$

With K: tensile strength coefficient, also known as the tensile strength determined by the Andreau formula [14]:

(2) $$K=10\;\left(\frac{20}{\rho^{0.64}}+\frac{V^{3.7}}{1294000\times \rho^{2.08}}\right)$$

To obtain data for the power dissipated using the dynamic resultant theorem [15,16], the following eq. is utilized.

(3) $$P_{m}=\frac{1}{\eta }\times \;v\;\times \;\left(M\times K\right)$$

At a maximum speed of 35.7 km/h and for the four (4) given cases, load is varied from 300 kg, 400 kg, 500 kg and 1000 kg mass, we note a difference. By averaging the different values of rolling resistance, we have Table 9.

**Table 9:** Rolling Resistance value using at a maximum speed for varying mass.

Maximum Speed *(km/h)*	mass *(kg)*	Rolling resistance*(N)*
	300	31.90
35.7	400	42.54
	500	53.17
	1000	106.35

**Fig. 11:**
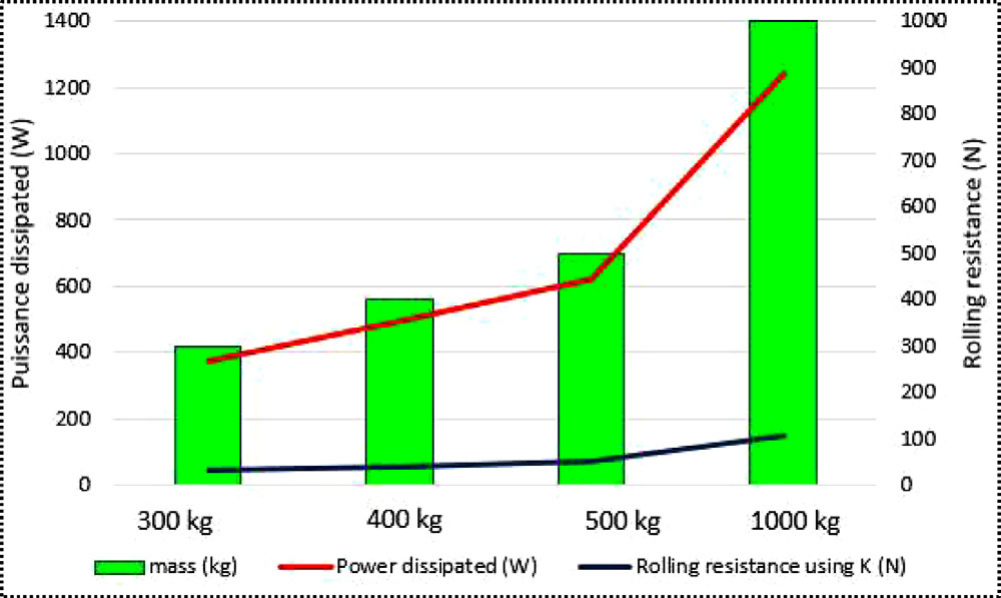
Curves for increasing performance and rolling resistance.

Generally speaking, the greater the load and the greater the friction generated by each wheel [17] and, consequently, the rolling resistance with values of 31.90 N for a 300 kg load, 42.54 N for 400 kg, 53.17 N for 500 kg and 106.35 N for 1000 kg. Fig. 11 illustrates that a minor fluctuation in rolling resistance leads to a significant rise in power dissipation.

### 4.3 Summary

This research is of great importance for the characterisation of these electrical vehicles, particularly those powered by photovoltaic solar energy like our vehicle and operating under the conditions examined. Additionally, it is essential for an energy modelling study of solar vehicles, as it explores the power dissipation caused by rolling resistance. It aims to provide objective information and establish causal links between technical variables. This study can be utilized for an electric vehicle that travels under equivalent test conditions and employs the identical type of motors utilized for the wheels. The motors used are Brushless BLDC motors which are brushless and therefore maintenance free. They have advantages, particularly in terms of efficiency [18]. They are capable of operating at 10,000 rpm under certain circumstances so it's important to, use this motor for electrical vehicle.

## Limitations

Not applicable.

## Ethics Statement

This data collection does not involve humans, animals, and is not sourced from social media networks.

## CRediT authorship contribution statement

**Sidy Mactar Sokhna:** Conceptualization, Methodology, Software, Writing – original draft. **Mohamed El Amine Ait Ali:** Visualization, Investigation, Writing – review & editing, Writing – original draft, Validation. **Souleye Faye:** Visualization, Validation. **Vincent Sambou:** Visualization, Validation. **Mohamed Agouzoul:** Visualization, Validation.

## Data Availability

Dataset on the power dissipated by rolling resistance of a prototype photovoltaic electric vehicle while varying the mass (Original data) (Mendeley Data).
